# PReS-FINAL-2222: FMF presenting with the features of malignancy

**DOI:** 10.1186/1546-0096-11-S2-P212

**Published:** 2013-12-05

**Authors:** G Keskindemirci, N Aktay Ayaz, D Tugcu, A Akcay, E Aldemir, G Aydogan

**Affiliations:** 1 Kanuni Sultan Suleyman Education and Research Hospital, Istanbul, Turkey

## Introduction

Familial Mediterranean fever (FMF) is an autoinflammatory disease characterized by periodic attacks of fever and serositis caused by mutations in FMF gene (MEFV). Splenomegaly and lymphadenopathy has been reported in FMF. The abdominal lymphadenopathy was reported in the mesentery during laparotomies for acute abdominal attacks of FMF.

## Objectives

A 14 year-old girl was admitted to the hospital with the complaints of fever, fatique and weight loss of 12 kg in 2 months duration. She had been prescribed different antibitiocis for fever. Her laboratory work-up was as follows; Hb. 7.4 gr/dl, Hct 23%, Wbc 6000/mm^3^, Plt 231000/mm^3^, ESR: 112 mm/hr, C-reactive protein: 52 mg/l, differential count and bone marrow aspiration was normal. Vitamin B12 level was low. Autoantibodies and microbiological work-up were unremarkable. She had hyperglobulinemia. Abdominal ultrasound revealed mild hepatosplenomegaly, but this was not noticed at physical examination. Pericardial effusion of 7 mm was present at echocardiography. Abdominal MRI revealed lymphadenopathy at paraaortic region and and splenic hilus. Positron emmision tomography was performed and increased fdg involvement at paraaortic, splenic and hepatic region, hypermetabolism at malignancy level and hypermetabolism in the spleen were detected. With the possible diagnosis of lymphoproliferative disease involving the spleen, an excisional biopsy was planned. During evaluation, the patient developed arthritis at her wrists. Due to the presence of fever, pericardial effusion, splenomegaly, arthritis and high inflammatory markers; MEFV mutation analysis was done. But in a month time, she lost 5 more kg, so a laparatomy and excisional biopsy was performed. Histopathology revealed only reactive lymphadenopathy without any malignant infiltration. She was found to be homozygous for M694V mutation. Colchicine tretament was introduced and nearly in a month time her ESR level decreased to 50 mm/hr and she had started to gain weight. In the next month's visit all of her complaints were gone and ESR became normal. She is still under colchicine treatment without any complication for 3 months.

## Conclusion

This is a very interesting FMF case presenting with the symptoms of malignancy and we were obliged to have a biopsy in order to exclude malignancy. In the literature there are few reports about such severe cases involving abdominal lymph nodes. This case is presented due to its unusual severe presentation and excellent response in 2 months time to colchicine.

## Disclosure of interest

None declared.

